# Unusually Large Ligand Field Splitting in Anionic Europium(III) Complexes Induced by a Small Imidazolic Counterion

**DOI:** 10.1021/acs.inorgchem.4c02729

**Published:** 2024-08-28

**Authors:** Lucca Blois, Israel F. Costa, João Honorato, Adalberto V Sanches de Araújo, Rômulo A. Ando, Albano N. Carneiro Neto, Markus Suta, Oscar L. Malta, Hermi F. Brito

**Affiliations:** † Department of Fundamental Chemistry, Institute of Chemistry, University of São Paulo, São Paulo 05508-000, Brazil; ‡ Physics Department and CICECO—Aveiro Institute of Materials, University of Aveiro, Aveiro 3810-193, Portugal; § Inorganic Photoactive Materials, Institute of Inorganic Chemistry, Heinrich Heine University Düsseldorf, Universitätsstr. 1, 40225 Düsseldorf, Germany; ∥ Department of Fundamental Chemistry, Federal University of Pernambuco, Recife 50740-560, Brazil

## Abstract

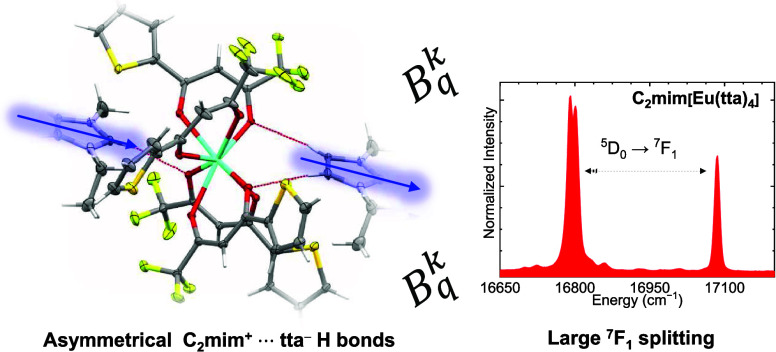

Luminescent trivalent lanthanide (Ln^3+^) complexes are compounds of technological interest due to their unique photophysical properties, particularly anionic *tetrakis* complexes, given their higher stability and emission quantum yields. However, structural studies on the cation–anion interaction in these complexes and the relation of such to luminescence are still lacking. Herein, the cation–anion interactions in two luminescent anionic tetrakis(2-thenoyltrifluoroacetonato)europate(III) complexes with alkylimidazolium cations, specifically 1-ethyl-3-methylimidazolium and 1-butyl-3-methylimidazolium are investigated. The Eu^3+^ complexes were synthesized and characterized by elemental analysis, mass spectrometry, and single-crystal X-ray crystallography, and their luminescence spectra were recorded at 77 K. Quantum chemical calculations were also performed. X-ray crystallography revealed hydrogen bonds between the enolate ligands and imidazolium ring hydrogens. The 1-butyl-3-methylimidazolium complex had two crystallographic Eu^3+^ sites, also confirmed by luminescence spectroscopy. The 1-ethyl-3-methylimidazolium complex exhibited an unusual 300 cm^–1^ splitting in the ^5^D_0_ → ^7^F_1_ transition, as reproduced by ligand field calculations, suggesting a stronger hydrogen bonding due to the smaller substituent. We hypothesize that this strong bonding likely causes angular distortions, resulting in high ligand field splittings.

## Abstract 2

This work explores the luminescence and structure of organic salts of an anionic tetrakis(enolato)europate(III) complex with 1-alkyl-3-methylimidazolium cations and 2-as thenoyltrifluoroacetonate (alkyl: ethyl and butyl, C*_n_*mim[Eu(tta)_4_]). Both complexes presented cation−anion hydrogen bonds. The C_4_mim[Eu(tta)_4_] presented two independent [Eu(tta)_4_]^−^ symmetrically H-bonded to the cation. C_2_mim[Eu(tta)_4_] was asymmetrically bonded to the cations. The C_2_mim[Eu(tta)_4_] presented an unusually large ligand field spitting of the ^7^F_1_ level, which we relate to the anisotropy generated by the asymmetric hydrogen bonds.

## 1 Introduction

Lanthanide complexes have been extensively studied in the field of luminescence due to the unique photophysical properties of the lanthanide ions (Ln^3+^), such as their narrow emission bands and relatively long emission lifetimes.^1−6^ In this context, many organic ligands (L) have been exploited in the synthesis of coordination compounds such as carboxylates,^7^ carbazolates,^8^ and β-diketonates.^9−11^ Each class of ligands has its peculiarities, such as solubilities, stability constants, and energy level structures. In the case of the Eu^3+^ ion, the β-diketonates have been employed for more than 50 years due to their efficient ligand-to-metal energy transfer to the Eu^3+^ ion.^12,13^

The Ln^3+^ ion possesses, however, a major drawback given that the 4f^*n*^–4f^*n*^ transitions are forbidden by the electric dipole (ED) mechanism since their Δ*l* = 0, which formally makes the intraconfigurational 4f transitions magnetic dipole (MD) allowed according to quantum mechanics. However, the intensity of MD transitions is by a factor of 10^5^ (1/4 α^2^ with α as the electromagnetic fine structure constant) lower in intensity than pure ED transitions. The fact that some of the experimentally observed transitions of the trivalent lanthanide ions still had higher intensities than expected for simple MD transitions puzzled researchers until the 1960s when Judd^14^ and Ofelt^15^ first described the relaxation of the ED selection rules due to the perturbation caused by the crystal field, which led to a mixing of the odd-parity 4f and even parity (5d, 6d, 5g, ···) configurations. However, this forced electric dipole (FED) mechanism is only responsible for a small fraction of the radiative rates of Eu^3+^ complexes.

After Judd–Ofelt works, the interaction between the exciting radiation and the ligand polarizabilities was also taken into account, a theoretical approach that is nowadays referred to as the dynamic coupling (DC) mechanism.^16−21^ The 4f^*n*^–4f^*n*^ transitions are allowed by the DC mechanism, but the order of magnitude for the radiative rates obtained from this mechanism is around that of the Judd–Ofelt theory (*A*_rad_ ≅ 10^2^ – 10^3^ s^–1^).

To overcome the low oscillator strengths of the 4f^*n*^–4f^*n*^ transitions and obtain lanthanide complexes with high brightness (defined as the extinction coefficient times the quantum yield, *B* = ε × ϕ_*Ln*_^*L*^^22^), organic ligands that can transfer the absorbed energy to the Ln^3+^ are employed in the synthesis of their compounds.^23^ The phenomenon of intramolecular energy transfer (IET) was first described by Weissman in 1942, observing the narrow 4f^6^–4f^6^ emission of the Eu^3+^ ion when excited into the organic ligand absorption band.^24^ Since the extinction coefficient of the Ln^3+^ ions is usually in the order of 1 L·mol^–1^·cm^–1^, the coordination of a ligand with high absorptivity (usually between 10^3^ and 10^4^ L·mol^–1^·cm^–1^) can lead to brightness thousands of times higher than those of Ln^3+^ compounds with inorganic ligands such as chlorides or nitrates.

The theoretical foundations and modeling for the energy transfer in Ln^3+^ coordination compounds have seen a major advance in the last three decades.^13,25−27^ Nowadays, two main mechanisms are thought to be responsible for the IET processes, them being the *exchange mechanism* and the *multipolar interaction*, leading to typical IET rates between 10^6^ and 10^8^ s^–1^.^28^ It is worth mentioning that in the IET theory, there are selection rules in the total angular momentum *J* of the lanthanide ion states, namely, |Δ*J*| = 0 or 1 (except in the case where *J* = *J*′ = 0) for the exchange mechanism and *J* + *J*′ ≥ λ ≥ |Δ*J*|, λ = 2, 4, or 6 for the multipolar interaction.^28^

In the context of the europium β-diketonate complexes, the 2-thenoyltrifluoroacetone (Htta) ligand comes into the spotlight as one of the majorly studied ligands in hydrated *tris* [Eu(tta)_3_(H_2_O)_2_], substituted *tris* [Eu(tta)_3_(L)*_n_*], and *tetrakis* complexes Q[Eu(tta)_4_] (Q^+^ is a monovalent counterion).^29−35^ These complexes have been applied to many areas, including triboluminescent crystals, doped polymers, and organic light-emitting diode (OLED) prototypes.^11,36−38^ The advantage of *tetrakis* complexes is that the only acceptor for the energy transfer process is the Ln^3+^ ion, as well as the absence of water molecules in the first coordination sphere that can act as efficient quenchers for the luminescence process.^39,40^ However, the interaction in the solid state of the Q^+^ cations in the *tetrakis* complexes with the lanthanide anion [Eu(tta)_4_]^−^ has been poorly studied, and the 4f states splitting is strongly dependent on the countercation, as it has been shown for the emission spectra of several Eu^3+^ complexes in previous works.^34,35,41−43^

Herein, we present the synthesis, characterization (including high-resolution mass spectrometry), single-crystal structure, and photoluminescence properties of the C_n_mim[Eu(tta)_4_] (C_2_mim: 1-ethyl-3-methylimidazolium, C_4_mim: 1-butyl-3-methylimidazolium) complexes in solid-state and solution phases. For the [Eu(tta)_4_]^−^ complex ion in solution, the geometry was optimized with density functional theory (DFT) calculations. Furthermore, we also present calculations of the *B*_*q*_^*k*^ parameters of the ligand field Hamiltonian and relate them to the splitting of the ^7^F*_J_* levels of the europium(III) ion. The target of our study is to investigate the effect of the intermolecular interaction between the cation and the complex anion on the luminescence properties of the coordination compounds in both solid-state and solution phases.

## 2 Methods

### 2.1 Experimental Procedures

The reagents 2-thenoyltrifluoroacetone (Htta, 99%), 1-ethyl-3-methylimidazolium chloride (C_2_mimCl, >98%), and 1-butyl-3-methylimidazolium chloride (C_4_mimCl, 99%) were all purchased from Sigma-Aldrich and used without further purification. Europium(III) oxide (Eu_2_O_3_, CSTARM 99.99%) was converted to Eu(NO_3_)_3_·5H_2_O through reaction with 68% nitric acid in distilled water.

The elemental analyses (CHN) were carried out on a PerkinElmer 2400 series II instrument, the thermogravimetric analyses (TGA) were measured using a TA Q500 thermoanalyser from 25 to 700 °C, and the mass spectrometry (electrospray ionization-high resolution mass spectrometry (ESI-HRMS)) data were recorded using the Bruker Daltonics Microtof (for the C_2_mim[Eu(tta)_4_]) and Bruker Daltonics Maxis 3G (for the C_4_mim[Eu(tta)_4_]) with a time-of-flight (TOF) detector. The electrosprays were generated using a 4.5 kV voltage and dried under a 4 L·min^–1^ dry *N*_2_(*g*) flux at 180 °C. The Raman spectra of the lanthanide organic salts were recorded in the solid state and in MeCN solution using a WITec Alpha300 Raman microscope equipped with a 20 mW HeNe laser (633 nm emission line) as the light source. The infrared absorption spectra (FTIR) were recorded in the solid state and with ATR configuration using a Bruker VERTEX 70v spectrometer under vacuum.

The emission and excitation spectroscopy measurements were performed on a Horiba Jobin-Yvon Fluorog-3 spectrofluorometer with a single excitation monochromator and an iHR320 emission monochromator. The excitation source was a 450 W xenon short-arc lamp, and the emission was detected using a Synapse CCD detector with 1024 × 512 pixels resolution for the emission and a photomultiplier tube for the excitation. The millisecond-range luminescence decay measurements were carried out using a pulsed Xenon short-arc lamp with a pulse width of less than 50 μs as an excitation source, and the emission was detected using a photomultiplier tube after a 100 μs delay from the pulse.

Single-crystal X-ray diffractions were performed at 100 K on a Rigaku Synergy-S diffractometer (HyPIX detector) with Mo Kα radiation (λ = 0.71073 Å). CrysAlisPro^44^ was used for data collection, cell refinement, data reduction, and multiscan method absorption correction. The structure was solved and refined using the software SHELXT2018 and refined by SHELXL2018 from the OLEX2 suite.^45^ All atoms, except hydrogen, were identified and refined by least-squares full matrix *F*^2^ with anisotropic thermal parameters. In both structures, the tta ligand displays disorder in the thiophene moiety, being refined with specific occupations in each case. Table S1 summarizes the main crystal data collections and structure refinement parameters, also including the CCDC deposit number for supplementary crystallographic data. The Hirshfeld surfaces were generated using Crystal Explorer 21.

The complexes were prepared following the standard procedure for the synthesis of these lanthanide(III) *tetrakis* complexes.^3,11,42,43,46^ 5 mmol of NaOH dissolved in 10 mL of distilled water were added to 5 mmol of Htta dissolved in 50 mL of 2-propanol under stirring at 60 °C. After ∼5 min, 1.5 mmol of 1-alkyl-3-methylimidazolium (alkyl: ethyl or butyl) chloride dissolved in 15 mL of 2-propanol was added to the main Na(tta) solution. In sequence, 1 mmol of Eu(NO_3_)_3_·5H_2_O dissolved in 15 mL of distilled water is added dropwise to the reacting mixture. After some minutes, a white crystalline powder precipitated out of the solution, and the reaction was carried out for 3 more hours to ensure completion. The product powder was filtered out, washed with cold ethanol, and then recrystallized from boiling 2-propanol (∼80 °C). The obtained crystals were used to perform CHN and thermogravimetric analyses to ensure purity. The crystals were also used to perform single-crystal X-ray diffraction, electrospray ionization high-resolution mass spectrometry (ESI-HRMS), Raman spectroscopy, and luminescence spectroscopy studies.

#### C_2_mim[Eu(tta)_4_]

ESI(+) MS: *m*/*z* C_2_mim^+^ = 111.0921 (calcd 111.0917), ESI(−) MS: *m*/*z* [^151^Eu(tta)_4_]^−^ = 1034.8693 (calcd 1034.8740). Elemental analysis for C_38_H_27_EuF_12_N_2_O_8_S_4%_found (calcd): C 39.77 (39.76), H 2.34 (2.37), N 2.44 (2.44).

#### C_4_mim[Eu(tta)_4_]

ESI(+) MS: *m*/*z* C_4_mim^+^ = 139.1233 (calcd 139.1230), ESI(−) MS: *m*/*z* [^151^Eu(tta)_4_]^−^ = 1034.8739 (calcd 1034.8740). Elemental analysis for C_40_H_31_EuF_12_N_2_O_8_S_4_%found (calcd): C 40.90 (40.86), H 2.63 (2.66), N 2.38 (2.34)

### 2.2 Theoretical Modeling

The ground-state geometry of the isolated [Eu(tta)_4_]^−^ complex ion in acetonitrile was optimized employing density functional theory (DFT) using the B3LYP functional^47,48^ with dispersion (D3) corrections^49^ and the def2-TZVPPD basis set^50,51^ to describe the organic ligand, and the MWB52 pseudopotential and basis valence set was used for the Eu^3+^ ion.^52,53^ We chose the B3LYP functional due to its success in obtaining good ground-state geometries for lanthanide complexes.^54,55^ The acetonitrile solvent was included using the implicit solvation through the conductor-like polarizable medium model (CPCM).^56^ The Raman spectra of the isolated anion [Eu(tta)_4_]^−^ and the two employed counterions (C_2_mim^+^ and C_4_mim^+^ cations) were calculated at the ground-state minima obtained by optimization at the same level of theory used before, with the theoretical values obtained for the frequencies been scaled by a factor of 0.968.^57^ All electronic structure calculations were done employing the Orca package (v5.0).^58^

The ligand field parameters (*B*_*q*_^*k*^) were calculated using the simple overlap model (SOM) developed by Malta,^59^ using a Python script developed by our group (for more details, see the SI). In the Wybourne notation,^60^ the ligand field Hamiltonian for an Ln^3+^ ion ([Xe]4f^*n*^) with a number *M* of ligating atoms in atomic units is given by (eq 1)
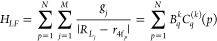
1where *C*_*q*_^(*k*)^ (*p*) is the spherical Racah tensor operator for the *p*th electron (defined as , with *Y*_*q*_^*k*^(θ_*p*_,ϕ_*p*_) being the spherical harmonic of rank *k* with the Condon–Shortley phase, *r*_4f_*p*__ is the position of the *p*th 4f electron, *R*_*L*_*j*__ is the position of the charge interacting with the 4f electron, and *g*_*j*_ is the charge factor of the ligating atom. In the SOM, the values of *R*_*L*_*j*__ are given by (eq 2)
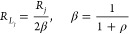
2with *R*_*j*_ being the position of the ligating atom, and ρ is the diatomic overlap integral between the Ln^3+^ atom and the ligating atom. For more details, see the original papers on the SOM.^59,61^

The ligand field parameters *B*_*q*_^*k*^ in atomic units (*E*_H_) are given by the following sum over the ligating atoms (eq 3):
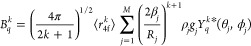
3where ⟨*r*_4f_^*k*^⟩ is the expectation value of the single-electron *r*_4f_^*k*^ operator. The diatomic overlap integrals were calculated using the equations reported elsewhere,^62^ which for the pair, Eu–O is given by ρ(*R*) = e^0.34–1.107*R*–0.074*R*^2^^. The complex and absolute values of the *B*_*q*_^*k*^ parameters are reported for positive values of *q* in the Supporting Information (Tables S2–S9). The *g*_*j*_ charge factor evaluation is the most challenging part of calculating the ligand field Hamiltonians. We have chosen to use the *g*_*j*_ values extracted from the best fit obtained from the *JOYSpectra program*(13) calculation of the theoretical 4f^*n*^–4f^*n*^ intensity parameters Ω_λ_ (λ = 2, 4, and 6) since they are unbiased with respect to the ligand field splitting.

For the C_4_mim[Eu(tta)_4_] compound, there are two Eu^3+^ crystallographic sites, and therefore, the experimental Ω_λ_ parameters have contributions from both sites. We have used the experimental intensity parameter values for both coordination polyhedra and used the fitted values of the charge factors (*g*_*j*_) from the *JOYSpectra program*.^13,63^

## 3 Results and Discussion

All of the characterization techniques (CHN, TGA, and ESI-HRMS) show the purity and stability of the prepared complexes. The ESI-MS spectra in the positive mode clearly show the C_*n*_mim^+^ cations, and in the negative mode, we have the [Eu(tta)_4_]^−^ anion with the characteristic isotopic pattern of the ^151/153^Eu isotopes (Figures S1 and S2).^41^ Furthermore, the thermogravimetric analyses (Figure S3) aside from showing that the complexes are air-stable until around 180 °C also indicate the absence of water molecules and, thus, confirm the tetrakis character of the investigated Ln^3+^ complexes.

**Figure 1:**
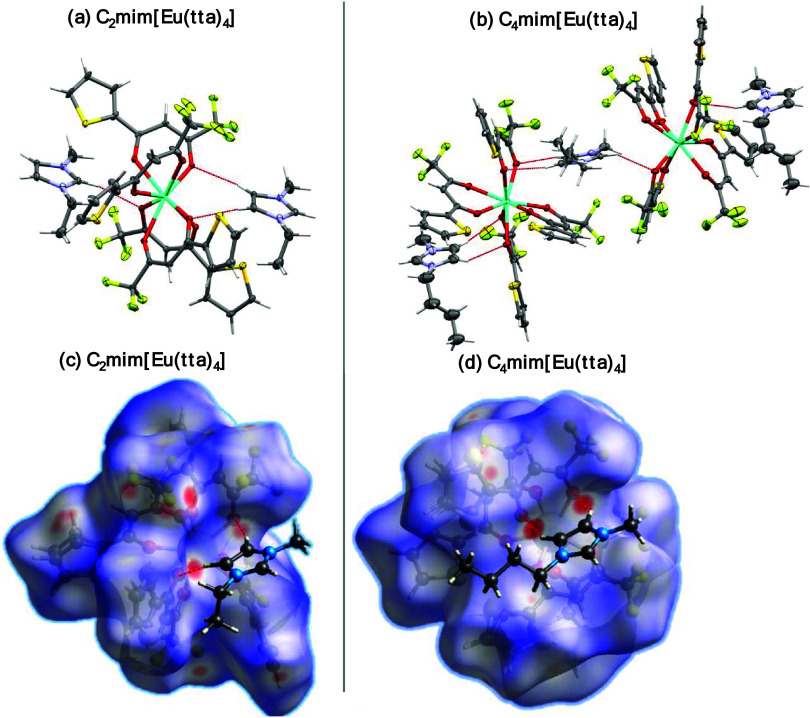
Crystallographic structures of (a) C_2_mim^+^[Eu(tta)_4_]^−^ and (b) C_4_mim^+^[Eu(tta)_4_]^−^ salts. Thermal ellipsoids were displayed with 30% probability. Hirshfeld surfaces for (c) C_2_mim^+^[Eu(tta)_4_]^−^ and (d) C_4_mim^+^[Eu(tta)_4_]^−^.

The intermolecular interactions in the solid state, determined by single-crystal X-ray diffraction, are driven by molecular packing in the *P*2_1_/*c* (no. 14) and *Cc* (no. 9) space groups for complexes C_2_mim^+^[Eu(tta)_4_]^−^ and C_4_mim^+^[Eu(tta)_4_]^−^, respectively. The crystallographic structures (Figure 1) show the C_2_mim^+^[Eu(tta)_4_]^−^ ionic network containing two molecules (one complex and one counterion) per asymmetric unity and the C_4_mim^+^[Eu(tta)_4_]^−^ with four molecules (2 complexes and 2 counterions). As shown in Figure 1, the obtained crystal structures display four bidentate tta ligands coordinated to the Eu^3+^ ion. Both crystal structures display a distorted square-antiprism geometry, as evidenced by the calculations performed by the Shape program (version 2.1), with the smallest continuous symmetry measures (CSM) values found between 0.3 and 0.5.

While the [Eu(tta)_4_]^−^ anionic unit with the C_2_mim^+^ cation not only has hydrogen bonds with the H(2) but also with H(4) and H(5) hydrogen atoms of the imidazolium ring, the interactions of the two nonequivalent [Eu(tta)_4_]^−^ anions with the C_4_mim^+^ cation are exclusive either with the H(4) and H(5) or the H(2) hydrogen atoms of the aromatic ring. Such hydrogen bonds have been known to occur in imidazolium ionic liquids^64−66^ and are responsible for some of their physical properties. In fact, the shorter H(2)–O distance of 2.39 Å in C_2_mim[Eu(tta)_4_] is just slightly longer than the distance of 2.23 Å calculated for the interaction of the [C_2_mim]^+^ cation with the triflate (CF_3_SO_3_^–^) anion, thus highlighting the presence of these interactions in our crystal samples.

Furthermore, most of the intermolecular interactions obtained from the Hirshfeld surface (Figure 1c,d) data correspond to weak interactions of the H···F type and nonclassical interactions of the F···F type. Although less numerous, the H···O interactions found correspond to the closer and consequently stronger interactions found, thus correlating to the hydrogen bonds present between C_*n*_mim^+^ cations and the [Eu(tta)_4_]^−^ complex anion.

C_2_mim[Eu(tta)_4_] has two bonding configurations of the tta ligand, as it was also observed for other asymmetric β-diketones *tetrakis* complexes similarly as in the Et_4_N[Eu(tta)_4_] (Et_4_N: tetraetylammonium),^37^ C_6_mim[Eu(tta)_4_],^46^ and the Bu_4_N[Eu(ntfa)_4_] (ntfa^–^: napthoyltrifluoroacetone) and C_4_mim[Eu(ntfa)_4_].^43^ Interestingly, the two coordination polyhedra of C_4_mim[Eu(tta)_4_] present the CF_3_/thiophene substituents grouped at one side of the polyhedron (Figure 1b).

**Figure 2:**
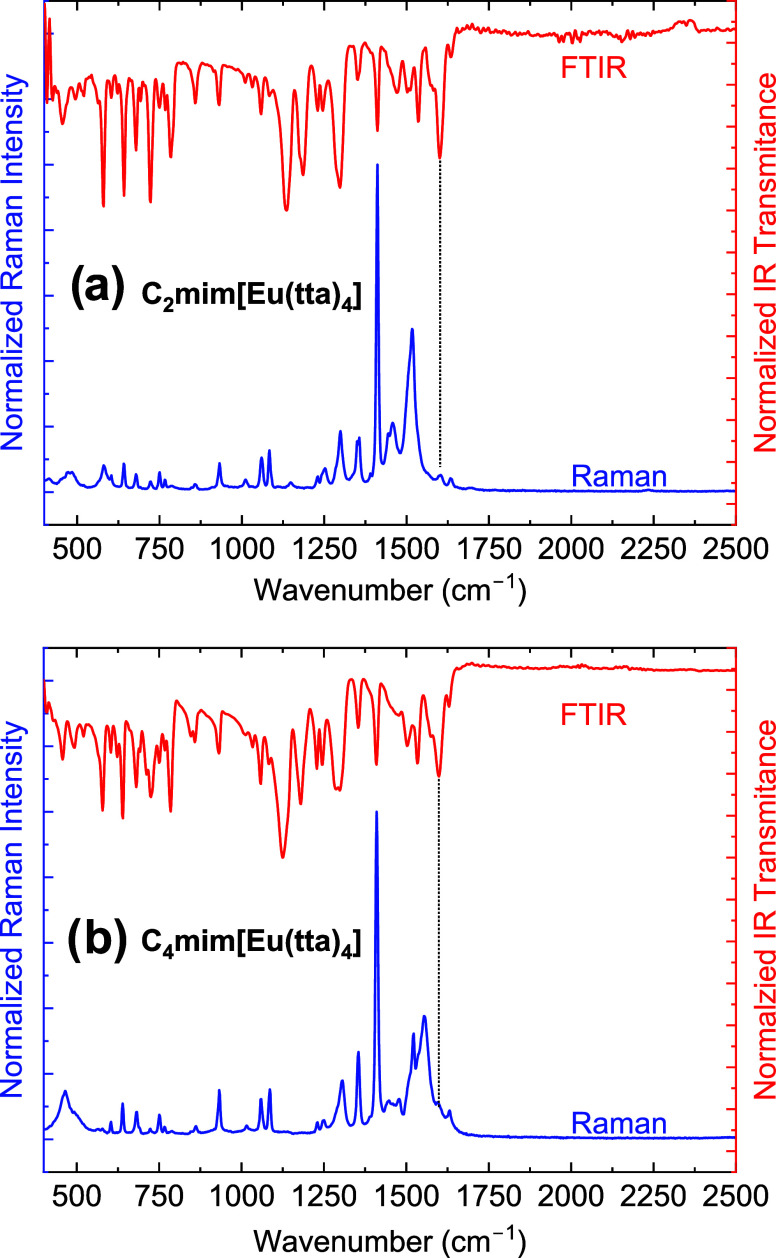
Raman (blue line) and FTIR-ATR (red line) spectra of (a) C_2_mim[Eu(tta)_4_] and (b) C_4_mim[Eu(tta)_4_] recorded in the solid state at 298 K. FTIR measurements were carried out under a vacuum. The dotted vertical line represents the carbonyl stretching frequency.

Besides correlating with the crystal structures, the Raman/FTIR spectra (Figure 2) of the organic salts show additional features for the C_4_mim^+^ cation (principally around 1500 cm^–1^), which we attribute to the two independent [Eu(tta)_4_]^−^ units.

The most intense band found in both Raman spectra can be assigned to the ν(C–S) from the thiophene ring around 1400 cm^–1^.^67,68^ The symmetric carbonyl stretching ν_s_(C=O) at 1598 cm^–1^ is in agreement with other europium(III) complexes with aromatic fluorinated β-diketonate ligands.^69−71^ Its relative intensity in the Raman spectra is low, as expected for C=O bonds compared to the C–C or C–S bonds. At around 1500 cm^–1^, the C–C=C enolate stretching can be seen with a high Raman intensity, which appears to be split in the C_4_mim[Eu(tta)_4_], probably due to the two crystallographic sites for the [Eu(tta)_4_]^−^ anion. Moreover, the vibrational mode containing the Eu–O stretching is present in the Raman spectra of both compounds at ∼480 cm^–1^.

**Figure 3:**
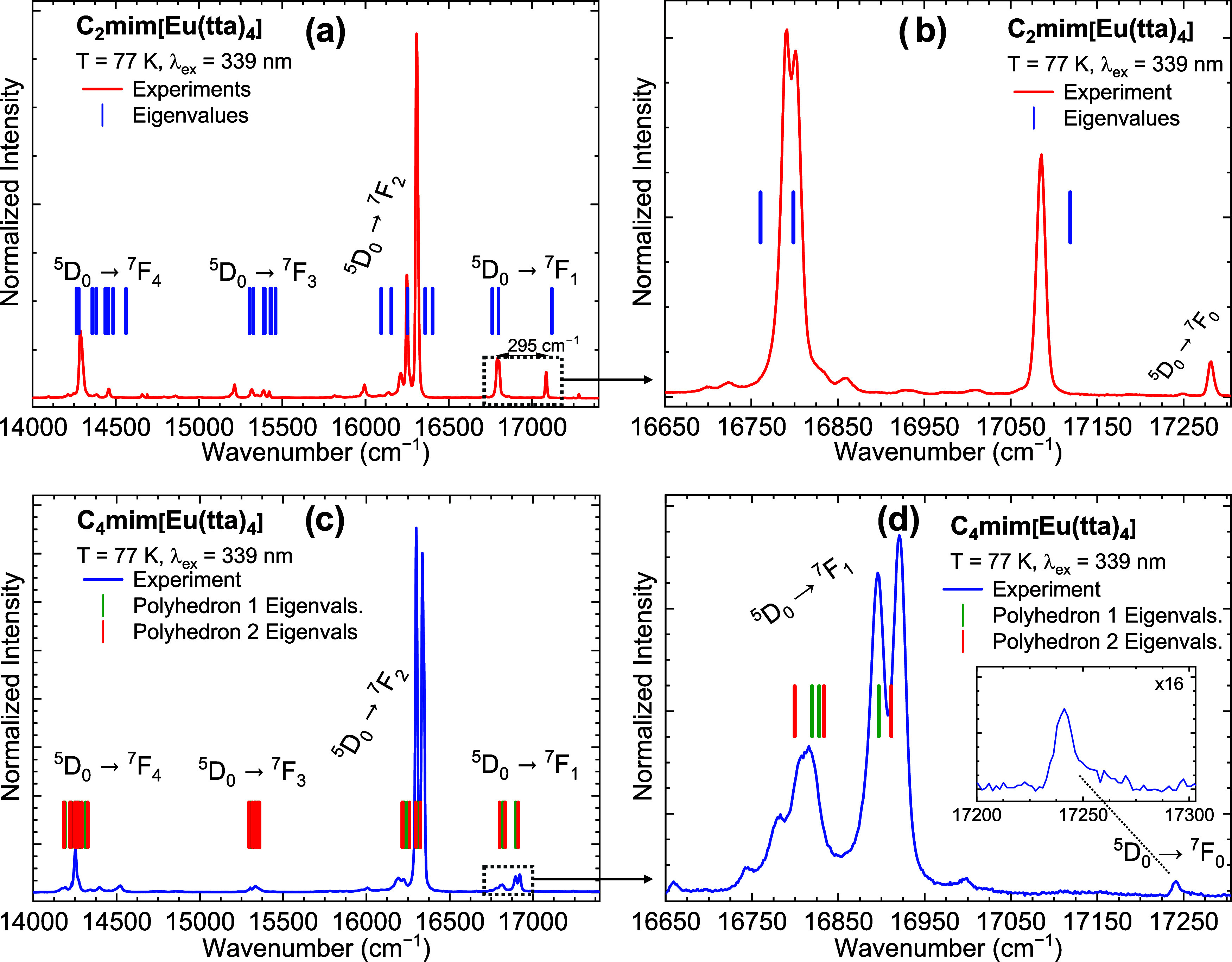
Emission spectra (a, c) of C*_n_*mim[Eu(tta)_4_] (*n* = 2 and 4) in the solid state at 77 K under ligand excitation at 339 nm and their calculated ligand field eigenvalues (vertical lines). (b, d) Zoom-in on the ^5^D_0_ → ^7^F_1_ transition with the calculated eigenvalues.

In the visible region, the emission spectra of the C_*n*_mim[Eu(tta)_4_] (*n* = 2, 4) salts (Figure 3a,c) show the characteristic ^5^D_0_ → ^7^F*_J_* (*J* = 0···6) narrow transitions of the europium(III) ion, with the most intense being the hypersensitive ^5^D_0_ → ^7^F_2_ transition at ∼614 nm. It is interesting to note the weak intensity of the ^5^D_0_ → ^7^F_0_ transition, which agrees with the distorted square-antiprism (*D*_4*d*_) structure of the coordination polyhedron observed in the crystallographic structures. It is noteworthy that this transition is symmetry-allowed only in the C_*n*_, C_*n*v_, and C_*s*_ point groups. A shoulder band can be observed on the ^5^D_0_ → ^7^F_0_ transition (∼17,250 cm^–1^) for C_4_mim[Eu(tta)_4_] (Figure 3d), which is consistent with the reported crystal structure presenting two similar albeit different [Eu(tta)_4_]^−^ anions.

**Figure 4:**
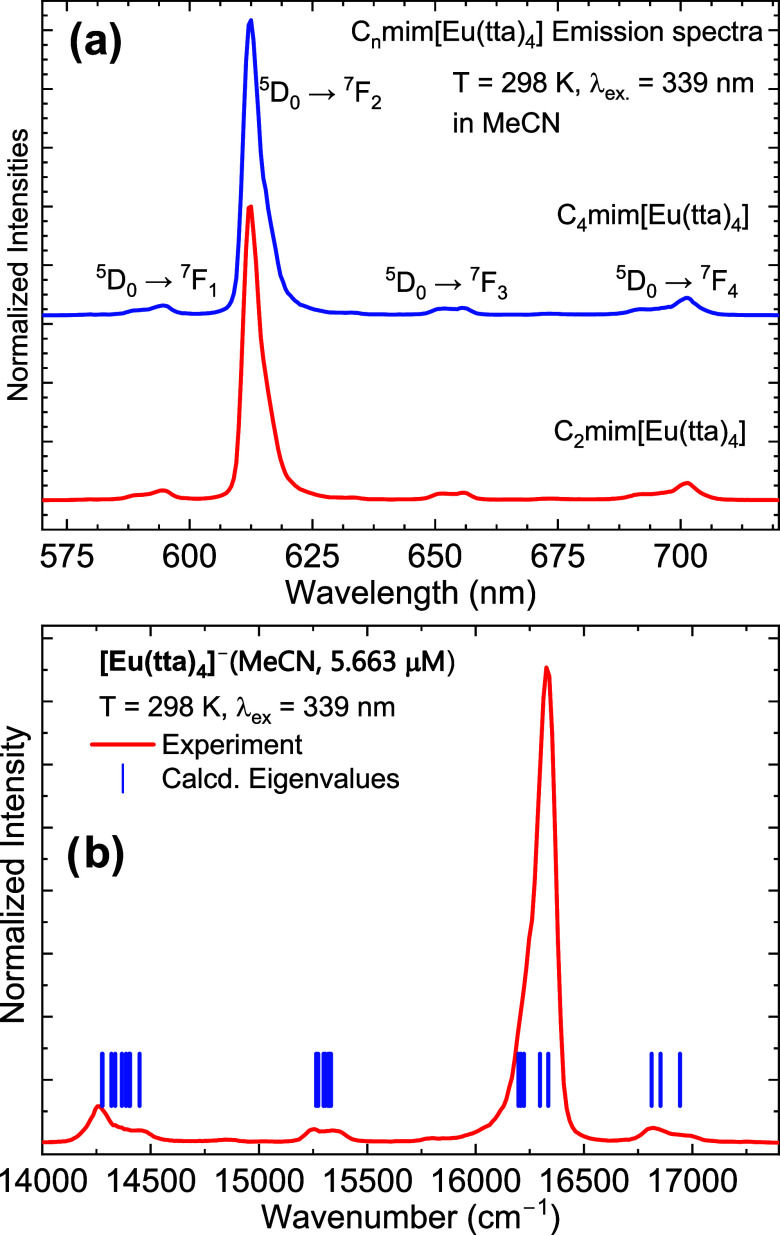
(a) Emission spectra (normalized) recorded in MeCN solutions of the C_2_mim[Eu(tta)_4_] (5.663 μM) and C_4_mim[Eu(tta)_4_] (5.315 μM) compounds at room temperature excited at the ligand band. (b) Calculated eigenvalues (blue ticks) were obtained using the SOM model.

It is possible to note the large splitting for the ^7^F_1_ level of the C_2_mim[Eu(tta)_4_] (Figure 3a,b) with an Δ*E* = 295 cm^–1^. Such a splitting is unusually large for Eu^3+^ chelates^72,73^ and is more often observed in ceramic Eu^3+^-based phosphors.^61,74^ For instance, one can infer a ∼190 cm^–1^ ligand field splitting of the ^7^F_1_ level from the emission spectrum reported in the literature for the stoichiometric *tris* complex 2-thenoyltrifluoroacetone ligand ([Eu(tta)_3_(H_2_O)_2_]).^2,75^ A recent study on the magnetism of Eu^3+^ compounds by Bronova and co-workers investigated the ligand field splitting in oxides and found most values in the range from 0 to 200 cm^–1^.^76^ In order to prove that this energy splitting is indeed from the ^7^F_1_ level, we calculated the theoretical ligand field parameters *B*_*q*_^*k*^ using the obtained crystallographic structure in the simple overlap model (SOM). One can then calculate the eigenvalues for the Stark energies of a ^7^F_*J*_ manifold with respect to the free ion by solving the secular determinant in degenerate first-order perturbation theory, neglecting any *J-mixing effect* (eq 4).
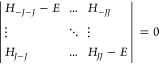
4where the matrix elements *H*_*mn*_ are given by (eq 5)
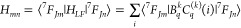
5With ^7^*F*_*Jm*_ representing an *M*_*J*_ = *m* state within the manifold. The matrix elements can be calculated using the Wigner–Eckart theorem (eq 6):
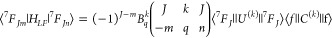
6where  is the Wigner 3*j* symbol,  is the reduced matrix element of the multielectron unitary tensor operator of rank *k*, and  is the reduced matrix element of the monoelectronic Racah operator of rank *k*. The reduced matrix elements then assume that the values of  and  are available in the Supporting Information (Table S10). Following the selection rules of the 3*j* symbol, the nonvanishing matrix elements for the ^7^F_1_ manifold are (eq 7)
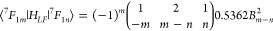
7We highlight that even though the *B*_*q*_^*k*^ parameters in the ligand field Hamiltonian are complex numbers, the relationship *Y*_*q*_^k*^ = (−1)^*q*^*Y*_–*q*_^*k*^ of the spherical harmonics ensure that *H*_*LF*_ forms a Hermitian matrix and, thus, the energy eigenvalues are always real. Using these matrix elements, we determined the secular determinant and calculated the ligand field splitting obtained from the crystallographic structures, including both possibilities for C_4_mim[Eu(tta)_4_]. Figure 4a,c shows the selected region of the ^7^F_1_ for both complexes together with the calculated eigenvalues (positioned with respect to the experimental transition centroid).

The abnormal LF splitting in the C_2_mim[Eu(tta)_4_] can be explained by the high absolute values of the *B*_0_^2^ and *B*_2_^2^ ligand field parameters (660.621 and 673.540 cm^–1^, respectively), while the low *B*_1_^2^ value (43.163 cm^–1^) gives rise to the small splitting of the doublet around 16,800 cm^–1^ (Figure 3a) and is associated with distortion from the ideal *D*_4_*_d_* symmetry. In C_4_mim[Eu(tta)_4_], the values for *B*_0_^2^ and *B*_2_^2^ are much smaller, with the largest being 188.007 cm^–1^.

Our current explanation for the large magnitude of the ligand field splitting occurring in the ^7^F_1_ energy level of the C_2_mim[Eu(tta)_4_] salt focuses mainly on the asymmetrical charge distribution around the Eu^3+^ ion. For the C_4_mim[Eu(tta)_4_] salt, both [Eu(tta)_4_]^−^ anions participate in hydrogen bonds with the same moiety of the imidazole ring (Figure 1b), as opposed to the C_2_mim[Eu(tta)_4_] compound, in which we have different portions of the imidazole ring participating in hydrogen bonds with the same complex anion. Such structural anisotropy leads to an angular charge asymmetry around the Eu^3+^ ion that reflects itself in the large values of the *B*_*q*_^2^ parameters. Indeed, the highest charge factor of 1.3 (Table S2) was observed for the O(5) atom, which engages directly into the shorter (and therefore stronger) H bond with the 2H from the imidazole ring. We hypothesize it to cause a sort of inductive effect on this tta ligand, as seen from the lower charge of 0.6 of the O(1) atom, belonging to the same tta ligand. That being, effects and variations on the crystal field parameters expressed in Wybourne’s notation are difficult to directly assign to a single factor as per their design that is comprised of a sum over the whole coordination polyhedron. In addition, we would like to predict that such a high magnitude of the ligand field splitting (Δ*E* = 295 cm^–1^) will induce an unusually strong van Vleck magnetic susceptibility due to the Boltzmann-populated A_2_ component split from the ^7^F_1_ level.

This intermolecular interaction of the organic cation with the first coordination sphere of the anion in the solid state is, however, not transposed to the solution medium. The room temperature spectra of the C*_n_*mim[Eu(tta)_4_] complexes (*n*: 2 and 4) in acetonitrile solution are identical (Figure 4a), suggesting that the solvated octa-coordinated anionic [Eu(tta)_4_]^−^ have essentially the same structure. Such a fact may be due to the high lability of Ln^3+^ ions, allowing the rearrangement of the ligands toward the thermodynamically more stable configuration.^77^ Given that the complexes are formally charged and acetonitrile is a relatively polar organic solvent (permanent dipole), it is to be expected that the C*_n_*mim^+^ cations and [Eu(tta)_4_]^−^ anions are to be solvated by MeCN molecules via ion-dipole and van der Waals interactions, thus decreasing (if not eliminating) the influence of the organic cation interaction with the first coordination sphere of the lanthanide ion. Furthermore, the room temperature spectra of the anionic [Eu(tta)_4_]^−^ complexes in solution are homogeneously broadened compared with the ones recorded for the solid compounds, which is a consequence of the more dynamic degrees of freedom of the complexes in solution.

The photophysical parameters of the Eu^3+^ ion derived from the 4f^*n*^–4f^*n*^ intensity theory such as the intensity parameters (Ω_2,4_) and the radiative rates (*A*_rad_) were calculated from the emission spectra. Together with the luminescence decay lifetime (Figure S4), the nonradiative decay rate (*A*_nrad_) and intrinsic emission quantum yield (ϕ_Ln_^Ln^) can be determined (Table 1). It is worth noting that even though the C_4_mim[Eu(tta)_4_] salt has two crystallographic sites for the Eu^3+^ ion, their characteristic decay rates could not be distinguished from the luminescence decay curves (Figure S4), although the two sites can still be distinguished by the ^5^D_0_ → ^7^F_0_ transition in the emission spectrum (Figure 3d), as corroborated by the Raman spectrum (Figure 2b).

**Table 1:** 4f–4f Intensity Parameters (Ω_2_, Ω_4_), Radiative (*A*_rad_) and Nonradiative (*A*_nrad_) Decay Rates, Lifetimes (τ), and Intrinsic Quantum Yields (ϕ_Eu_^Eu^) of the C*_n_*mim[Eu(tta)_4_] Complexes (*n* = 2 and 4)

complex^a^	Ω_2_ (10^–20^ cm^2^)	Ω_4_ (10^–20^ cm^2^)	*A*_rad_ (s^–1^)	*A*_nrad_ (s^–1^)	τ (μs)	ϕ_Eu_^Eu^ (%)
C_2_mim[Eu(tta)_4_]_(s)_	15.5 ± 0.5	9.28 ± 0.60	650 ± 16	698 ± 19	742	48.2 ± 1.3
C_4_mim[Eu(tta)_4_]_(s)_	33.1 ± 1.6	7.10 ± 1.39	1153 ± 54	326 ± 55	676	78.0 ± 3.7
[Eu(tta)_4_]_(MeCN)_^–^	49.3 ± 3.3	8.95 ± 0.74	1187 ± 70	445 ± 71	613	72.7 ± 4.7

a (s) denotes solid and (MeCN) acetonitrile solution.

These photophysical parameters were calculated using the procedure described in the Supporting Information. The lower Ω_2_ values compared to the other complexes (Table 1) show that with a small side-chain cation such as the C_2_mim^+^, the [Eu(tta)_4_]^−^ complex ion is in a more symmetrical structure when compared with the C_4_mim[Eu(tta)_4_] system. The increased radiative rate in the C_4_mim[Eu(tta)_4_] leads to a higher emission quantum yield, as well as a decrease in the nonradiative rate, which may be due to the weaker coupling to C–H oscillators. It is possible to see through the intensity parameters that the coordination environment around the Eu^3+^ ion is further distorted from the respective solid states when it is dissolved in the MeCN solvent, as anticipated by the increased value of the Ω_2_ parameter.

The density functional theory (DFT) ground-state geometry optimized with implicit solvation (CPCM) at the B3LYP/def2-TZVPPD/MWB52(Eu) level of theory also displays a square antiprismatic geometry around the Eu^3+^ ion. It is noteworthy that even though the C_2_mim[Eu(tta)_4_] and C_4_mim[Eu(tta)_4_] compounds have different arrangements regarding the CF_3_ and thiophene rings of the ligands, they should behave similarly in solution, and due to homogeneous broadening, they present virtually the same emission spectrum. Furthermore, the fact that the luminescence decay curves recorded in solution from both complexes have the same decay constant together with the high lability of Ln^3+^ ions indicates that there is only one *de facto* emitting species in solution.

**Figure 5:**
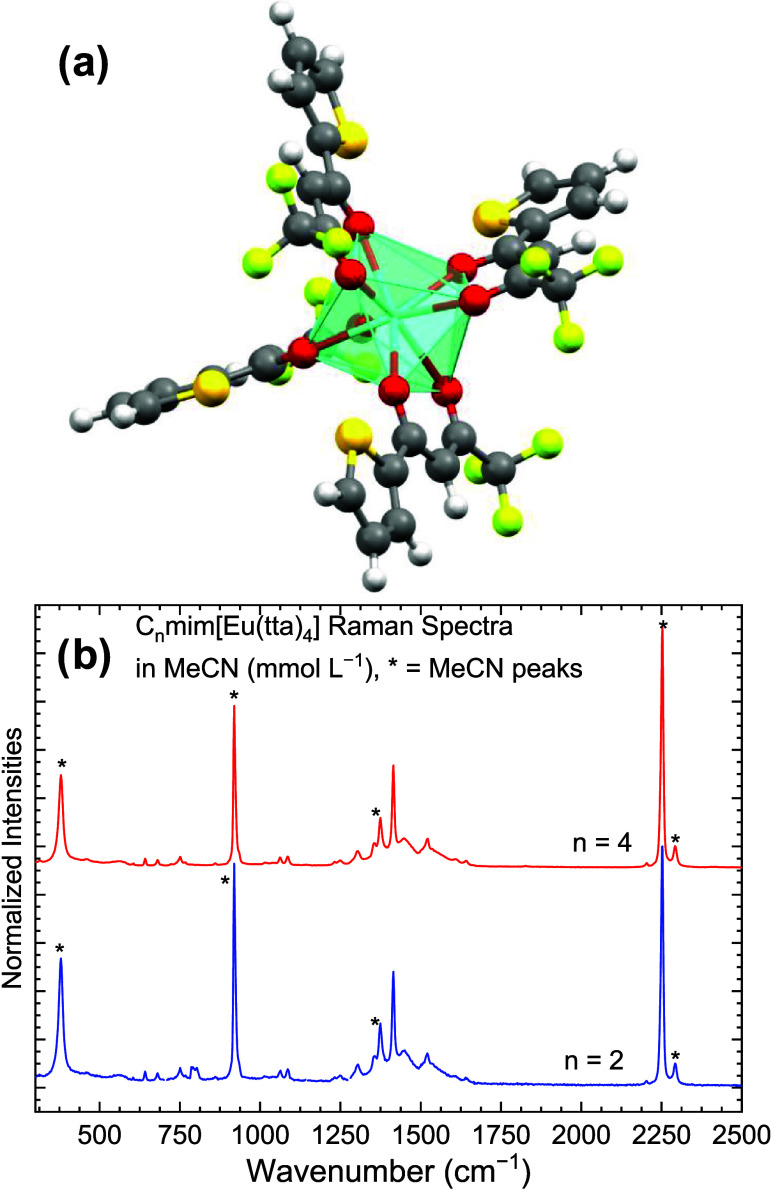
(a) Ground-state geometry optimized for the [Eu(tta)_4_]^−^ in acetonitrile solution at the B3LYP/def2-TZVPPD/MWB52(Eu)/CPCM(MeCN) level of theory. (b) Experimental Raman spectra of the solution of both complexes.

The Raman spectra obtained in MeCN (Figure 5a,b) solution with both cations show similar vibration features, with the most relevant scattering bands appearing at the region between 1220 and 1700 cm^–1^, where the band at 1415 cm^–1^ shows the highest intensity. The main difference between the spectra is observed around 800 cm^–1^, in which the C_2_mim[Eu(tta)_4_] spectrum presents a low-intensity band that is absent for the C_4_mim[Eu(tta)_4_] spectrum. The calculated Raman spectrum (Figure S5) indicates that both the cations and the [Eu(tta)_4_]^−^ complex present intense bands at 1220 and 1700 cm^–1^, with the spectra of both cations appearing identical. It is challenging to quantify the distinct contributions of the cations to the Raman spectra as they present similar peak structures in this region.^78^ Nevertheless, the calculated spectra present a major shift of 2 cm^–1^ between the bands at 1355 cm^–1^ when compared to the experimental ones, and we can suppose that the spectra are dominated by the contributions of the Eu^3+^ complex anion with the majority of the bands possibly being assigned to the internal modes of the tta ligands. As it can be seen, the band at 1415 cm^–1^ (calculated at 1398 cm^–1^) is mainly attributed to a C–C stretching plus in-plane bending mode of the thiophene ring (in coincidence with a ring mode of the imidazolium ring calculated at 1396 cm^–1^), and the band at 1449 cm^–1^ associated with the asymmetric stretches of the carbonyls bonded to the metal ion (calculated 1438 cm^–1^).

The *B*_*q*_^*k*^ ligand field parameters for the [Eu(tta)_4_]^−^ complex anion in an acetonitrile solution were also calculated using the SOM Hamiltonian (Table S8). By using the *g*_*j*_ values from the JOYSpectra program, we have obtained good agreement between the ligand field splitting (Δ*E*) and the experimental spectrum (Figure 4b), indicating that ground-state geometry from the DFT calculations satisfactorily describes the {EuO_8_} coordination polyhedron in solution. Furthermore, the full set of calculated eigenvalues describes the profile of the entire emission spectrum of the [Eu(tta)_4_]^−^ in solution (Figure 5b), suggesting that if one takes the homogeneous broadening caused by the MeCN solution into account, the calculated DFT structure can be a representative of the [Eu(tta)_4_]^−^ complex in solution.

The ligand field strength of the solvated [Eu(tta)_4_]^−^ in MeCN solution falls within the commonly observed range for europium(III) compounds (and closer to that of the C_4_mim[Eu(tta)_4_]),^79,80^ further indicating that the unusually large splitting (295 cm^–1^ for the ^7^F_1_ level) found for the C_2_mim[Eu(tta)_4_] is a result of the cation–anion interaction in the crystal phase, much possibly caused by the proximity of the [C_2_mim]^+^ counterion and hydrogen bonding with the H(2) atom from the imidazole ring.

## 4 Conclusions

We successfully managed to prepare the 1-ethyl-3-methylimidazolium and 1-butyl-3-methylimidazolium salts of the [Eu(tta)_4_]^−^ complex anion and characterized their composition through high-resolution mass spectrometry and single-crystal X-ray diffraction. The structures refined from the X-ray diffraction data revealed that the oxygen atoms from the tta ligands that are coordinated enolate groups also engage in hydrogen bonds with the H(2), H(4), and H(5) atoms of the imidazole ring, further stabilizing the organic salts. Furthermore, X-ray crystallography hinted at two different [Eu(tta)_4_]^−^ in the structure, which was further confirmed by FTIR/Raman vibrational spectroscopies and the Eu^3+^ emission from the C*_n_*mim[Eu(tta)_4_] compounds.

Curiously, the emission spectrum of C_2_mim[Eu(tta)_4_] showed an unexpectedly strong splitting of the ^7^F_1_ level (295 cm^–1^) due to ligand field interactions. Utilizing theoretical calculations of the ligand field parameters (*B*_*q*_^*k*^) via the simple overlap model (SOM), we satisfactorily reproduced the large splittings. Within the model, we can attribute the unexpected ligand field effect to a charge asymmetry in the coordination polyhedron that is most possibly caused by the presence of different hydrogen bonds with the organic cation (H(2) and H(4),H(5) atoms). This is in contrast to the two [Eu(tta)_4_]^−^ complexes in the C_4_mim^+^ salts, where each anionic complex has hydrogen bonds to the same part of the imidazole ring (*either* the H(2) *or* H(4), H(5) atoms) of the cation. Such is an interesting feature of the imidazolium cation salt of anionic lanthanide(III) complexes. We believe that these results can inspire follow-up studies, such as investigating this effect in the van Vleck magnetic susceptibility of such Eu^3+^-based complexes or the effect of even smaller cations in these systems.

## Supporting Information Available

Crystal data and structure refinement, ESI-MS spectra, TG curves and luminescence decay curves for all compounds, calculated ligand field parameters, equations for 4f–4f intensity parameters calculation, and theoretical Raman spectra (PDF)
