# Supplementary material for: Unusually Large Ligand Field Splitting in Anionic Europium(III) Complexes Induced by a Small Imidazolic Counterion
Source: Inorg Chem. 2024 Aug 28;63(36):16861–71. doi: 10.1021/acs.inorgchem.4c02729 (PMC11388464; doi:10.1021/acs.inorgchem.4c02729)
Supplement: Supplementary file 1 — ic4c02729_si_001.pdf [file ic4c02729_si_001.pdf]

Supporting Information

Unusually large ligand field splitting in anionic europium(III) complexes induced by a small imidazolic counterion

Lucca Blois[1], Israel F. Costa[1], João Honorato[1], Adalberto V. Sanches de Araújo[1], Rômulo A. Ando[1], Albano N. Carneiro Neto[2], Markus Suta[3], Oscar L. Malta[4], Hermi F. Brito[1*]

[1]*Department of Fundamental Chemistry, Institute of Chemistry, University of São Paulo, São Paulo, Brazil*

[2] *Physics Department and CICECO—Aveiro Institute of Materials, University of Aveiro, Aveiro, Portugal*

[3]*Inorganic Photoactive Materials, Institute of Inorganic Chemistry, Heinrich Heine University Düsseldorf, Universitätsstr. 1, 40225 Düsseldorf, Germany*

[4]*Department of Fundamental Chemistry – Federal University of Pernambuco, Recife, Brazil*

*e-mail: hefbrito@iq.usp.br

Supporting Information

Table S1. Crystal data and structure refinement for the synthetized salts $C_2mim[Eu(tta)_4]$ (CCDC 2352756) and $C_4mim[Eu(tta)_4]$ and (CCDC 2352757).

Identification code	$C_2mim^+[Eu(tta)_4]^-$	$C_4mim^+[Eu(tta)_4]^-$
CCDC code	2352756	2352757
Empirical formula	$C_{38}H_{27}EuF_{12}N_2O_8S_4$	$C_{40}H_{31}EuF_{12}N_2O_8S_4$
Formula weight	1147.81	1175.65
Temperature/K	100.00(17)	100.00(16)
Crystal system	monoclinic	monoclinic
Space group	$P2_1/n$	Cc
a/Å	10.72544(14)	10.41481(11)
b/Å	20.1122(3)	40.0711(6)
c/Å	20.2144(3)	22.5683(2)
α/°	90	90
β/°	93.1577(14)	103.0599(11)
γ/°	90	90
Volume/Å^3	4353.88(11)	9174.87(19)
Z	4	8
ρ_{calc}g/cm^3	1.751	1.702
μ/mm^{-1}	1.736	1.650
F(000)	2272.0	4671
Crystal size/mm^3	0.217 × 0.046 × 0.042	0.276 × 0.042 × 0.02
Radiation	Mo Kα (λ = 0.71073)	MoKα (λ = 0.71073)
2θ range for data collection/°	4.846 to 51.364	4.894 to 51.698
Index ranges	$-13 \leq h \leq 13$, $-24 \leq k \leq 24$, $-24 \leq l \leq 24$	$-12 \leq h \leq 12$, $-49 \leq k \leq 49$, $-27 \leq l \leq 27$
Reflections collected	50755	99376
Independent reflections	8263 [R_{int} = 0.0339, R_{sigma} = 0.0229]	17592 [Rint = 0.0623, Rsigma = 0.0335]
Data/restraints/parameters	8263/308/612	17592/772/1322
Goodness-of-fit on F^2	1.018	1.131
Final R indexes [I>=2σ (I)]	R_1 = 0.0285, wR_2 = 0.0654	R1 = 0.0482, wR2 = 0.1344
Final R indexes [all data]	R_1 = 0.0317, wR_2 = 0.0668	R1 = 0.0490, wR2 = 0.1365
Largest diff. peak/hole / e Å^{-3}	1.15/-0.69	1.38/-2.57

Supporting Information

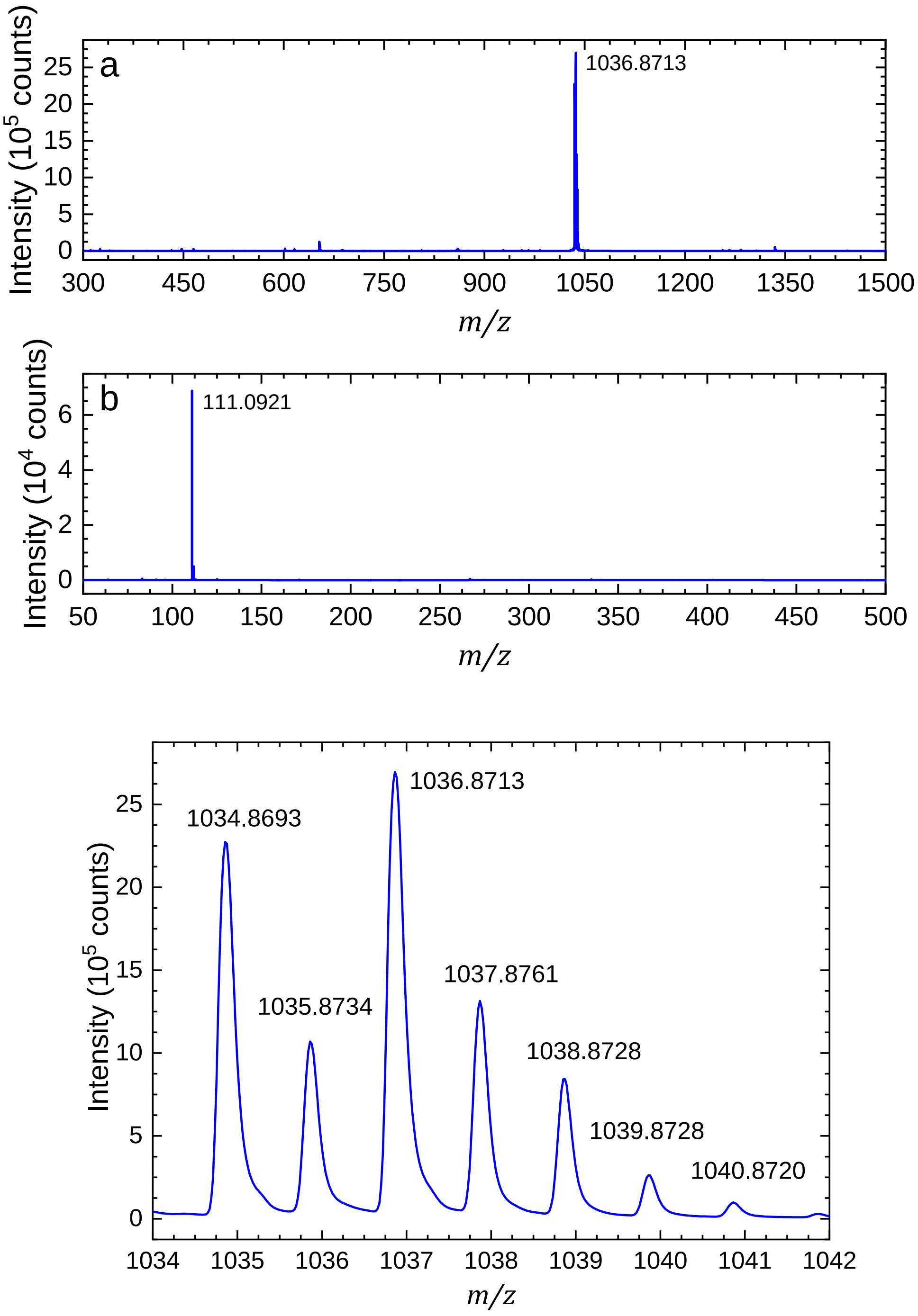

Figure S1. High resolution ESI spectrum for the C_2mim[Eu(tta)$_4$] complex in the (a) negative and (b) positive modes.

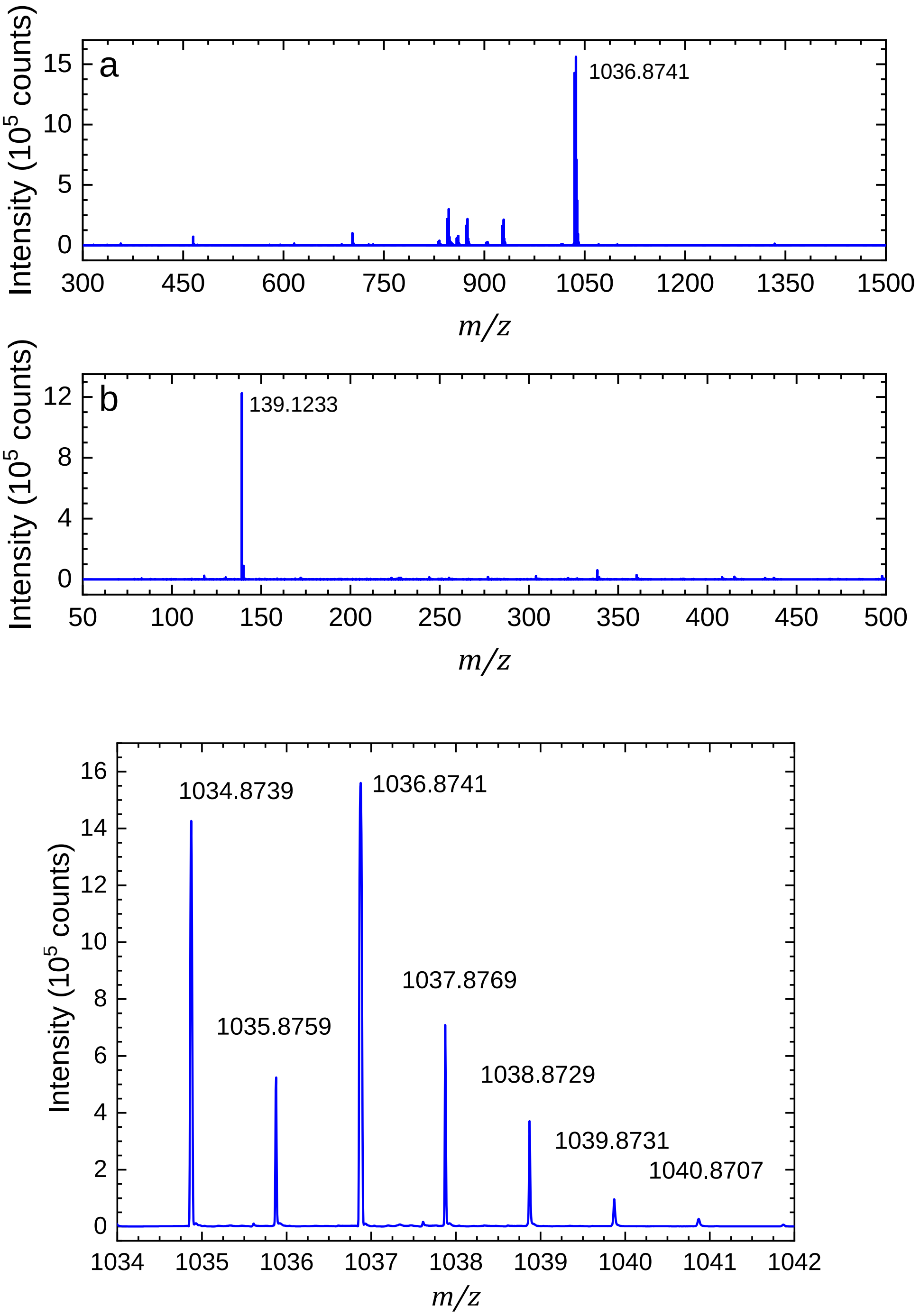

Figure S2. High resolution ESI spectrum for the C_4mim[Eu(tta)$_4$] complex in the (a) negative and (b) positive modes.

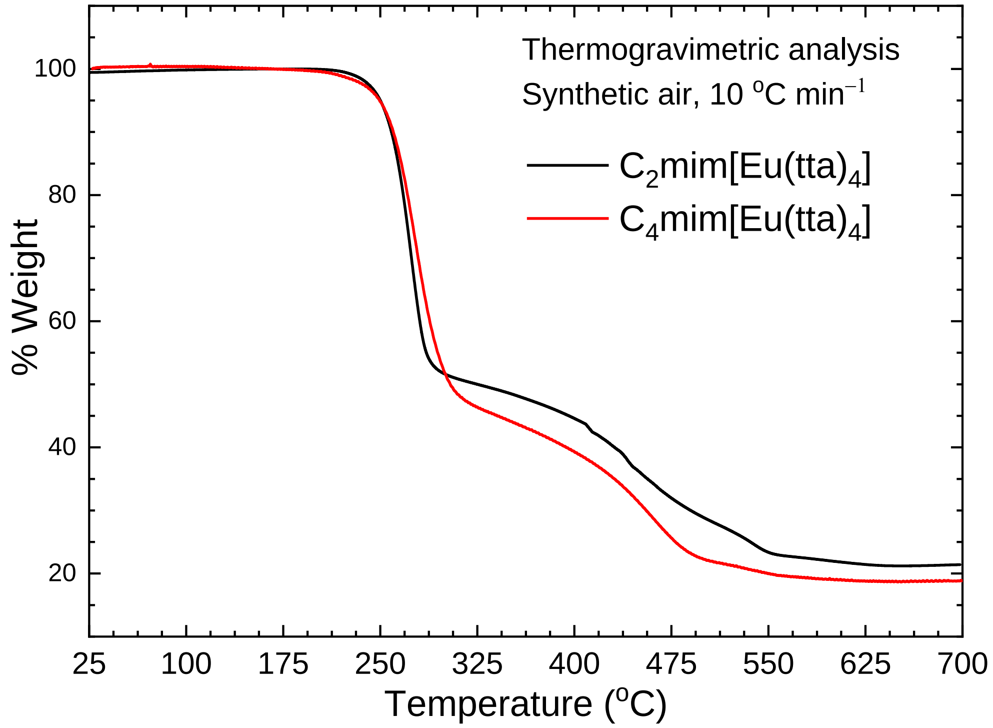

Figure S3. Thermogravimetric curves of the $C_n$mim[Eu(tta)$_4$] complexes recorded in synthetic air and 10 °C min^{-1} heating rate.

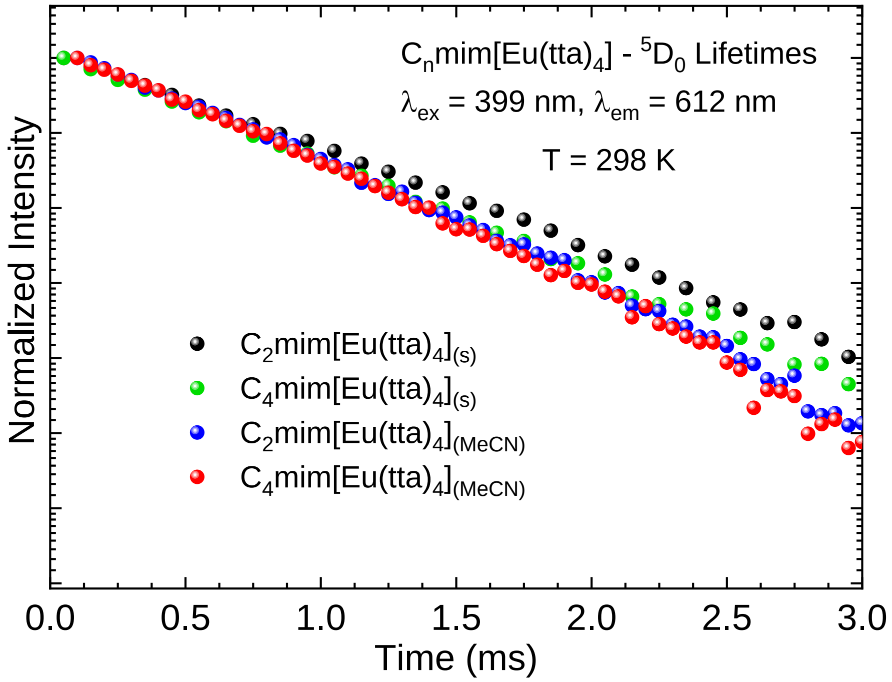

Figure S4. Luminescence decay curves of the $C_n$mim[Eu(tta)$_4$] complexes registered at room temperature (298 K) in solid state and acetonitrile (MeCN) solution with excitation at the ligand absorption band and monitoring the Eu^{3+} emission.

Supporting Information

The calculation of the ligand-field Hamiltonian, its matrix elements and the secular determinant were done using a Python script that reads the Geometry and Charge Factors of the atoms. The script is available for free through an AGPL-3.0 license at https://github.com/luccablois/SOLkq/blob/main/SOLkq.py

Negative q values not reported for clarity. They can be calculated through: $B_{-q}^{k} = (-1)^q B_q^{k^*}$

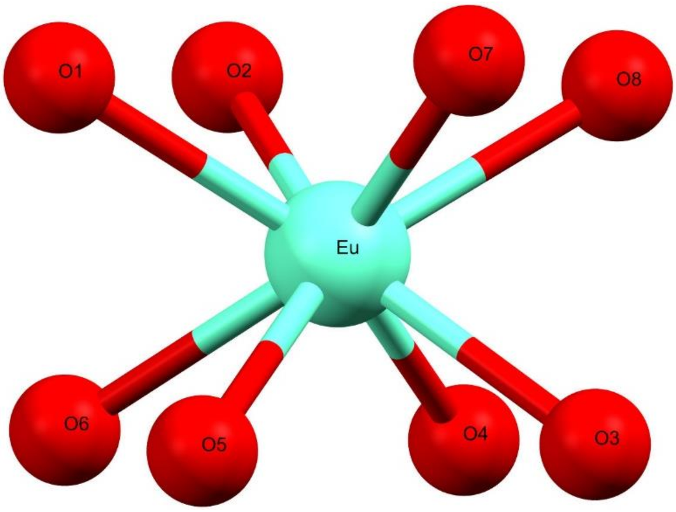

Table S2. g_j values obtained by the best JOYSpectra Ω_λ fit and overlap integrals ρ for the C_2mim[Eu(tta)$_4$].

Atom	g_j	ρ
O(1)	0.574	0.0646
O(2)	1.022	0.0656
O(3)	0.663	0.0657
O(4)	1.195	0.0672
O(5)	1.335	0.0646
O(6)	1.137	0.0662
O(7)	1.217	0.0663
O(8)	1.027	0.0677

Table S3. Ligand field parameters (B^k_q) for the C_2mim[Eu(tta)$_4$] complex.

Parameter	Complex value / cm^{-1}	Absolute value / cm^{-1}
B^2_0	$660.621 + 0i$	660.621
B^2_1	$27.682 - 33.117i$	43.163
B^2_2	$-493.347 - 458.546i$	673.540
B^4_0	$-362.852 + 0i$	362.852
B^4_1	$-352.737 + 190.741i$	401.006
B^4_2	$216.397 - 37.111i$	219.560
B^4_3	$227.218 - 190.399i$	296.446
B^4_4	$-236.012 + 334.988i$	409.780
B^6_0	$-118.238 + 0i$	118.238
B^6_1	$-124.273 + 11.767i$	124.829
B^6_2	$45.995 - 46.440i$	65.362
B^6_3	$336.835 - 89.735i$	348.583
B^6_4	$-169.842 + 99.066i$	196.623
B^6_5	$-109.499 + 224.006i$	249.337
B^6_6	$127.398 - 162.185i$	206.239

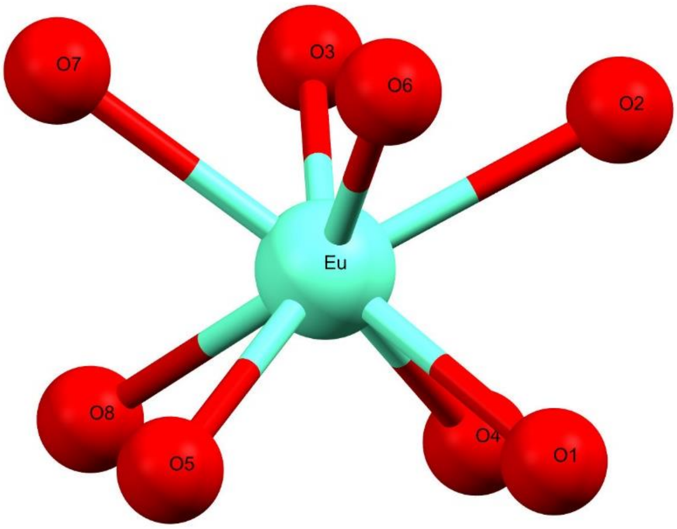

Table S4. g_j values obtained by the best JOYSpectra Ω_λ fit and overlap integrals ρ for the C_4mim[Eu(tta)$_4$] polyhedron 1.

Atom	g_j	ρ
O(1)	0.431	0.0652
O(2)	0.433	0.0630
O(3)	0.558	0.0676
O(4)	0.560	0.0665
O(5)	0.446	0.0672
O(6)	0.487	0.0662
O(7)	0.559	0.0634
O(8)	0.432	0.0657

Table S5. Ligand field parameters (B_q^k) for the C_4mim[Eu(tta)$_4$] complex polyhedron 1.

Parameter	Complex value / cm^{-1}	Absolute value / cm^{-1}
B_0^2	$45.662 + 0i$	45.662
B_1^2	$-56.555 + 102.322i$	116.911
B_2^2	$96.742 + 85.198i$	128.910
B_0^4	$-134.102 + 0i$	134.102
B_1^4	$33.734 - 166.358i$	169.746
B_2^4	$-118.258 - 9.253i$	118.620
B_3^4	$61.228 - 94.549i$	112.643
B_4^4	$-260.629 - 12.862i$	260.947
B_0^6	$27.480 + 0i$	27.481
B_1^6	$32.328 - 77.066i$	83.572
B_2^6	$-52.235 - 5.076i$	52.481
B_3^6	$11.953 - 69.529i$	70.549
B_4^6	$-78.350 + 2.691i$	78.397
B_5^6	$25.324 - 144.270i$	146.476
B_6^6	$-90.937 - 47.614i$	102.648

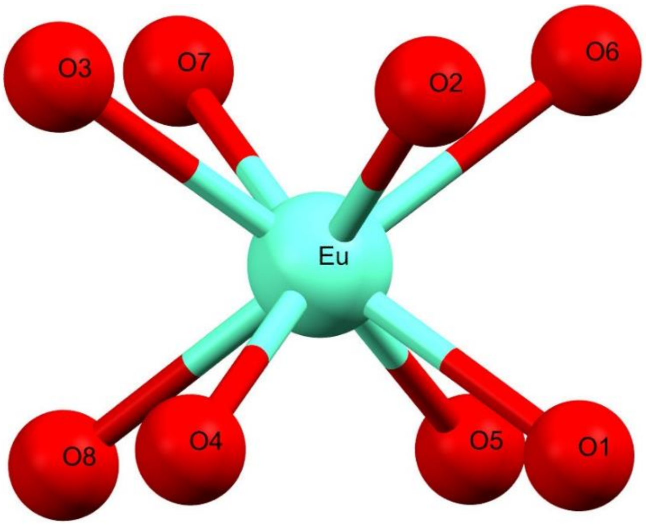

Table S6. g_j values obtained by the best JOYSpectra Ω_λ fit and overlap integrals ρ for the C_4mim[Eu(tta)$_4$] polyhedron 2.

Atom	g_j	ρ
O(1)	0.495	0.0655
O(2)	0.414	0.0660
O(3)	0.541	0.0657
O(4)	0.623	0.0671
O(5)	0.551	0.0674
O(6)	0.412	0.0630
O(7)	0.549	0.0666
O(8)	0.441	0.0659

Table S7. Ligand field parameters (B_q^k) for the C_4mim[Eu(tta)$_4$] complex polyhedron 2.

Parameter	Complex value / cm^{-1}	Absolute value / cm^{-1}
B_0^2	$-138.546 + 0i$	138.546
B_1^2	$-88.679 + 67.179i$	111.252
B_2^2	$171.159 + 77.789i$	188.007
B_0^4	$-174.441 + 0i$	174.441
B_1^4	$20.862 + 205.993i$	207.047
B_2^4	$-33.256 - 46.003i$	56.765
B_3^4	$14.624 + 199.295i$	199.831
B_4^4	$-209.945 + 16.791i$	210.616
B_0^6	$2.102 + 0i$	2.102
B_1^6	$-18.181 + 13.453i$	22.618
B_2^6	$21.183 + 7.719i$	22.546
B_3^6	$-0.324 + 202.738i$	202.738
B_4^6	$-63.065 - 8.968i$	63.700
B_5^6	$37.416 - 84.332i$	92.260
B_6^6	$-121.567 - 21.419i$	123.441

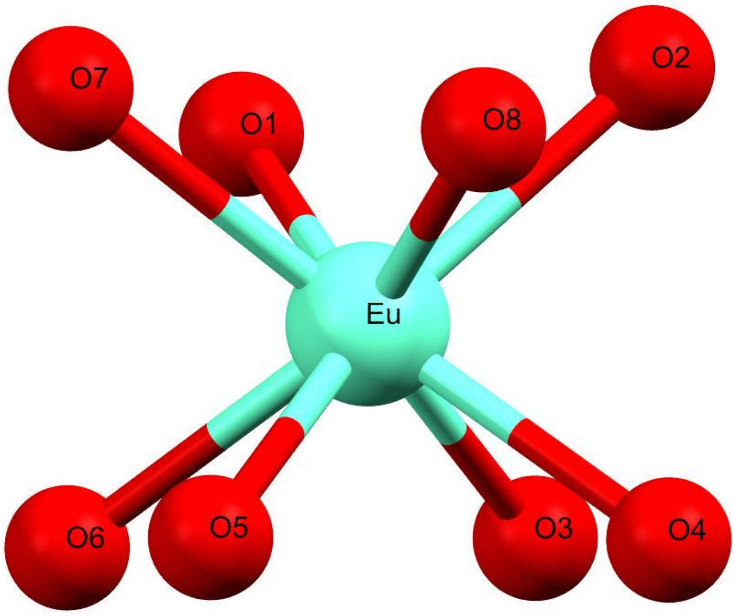

Table S8. g_j values obtained by the best JOYSpectra Ω_λ fit and overlap integrals ρ for the $[Eu(tta)_4]^-$ ground state geometry obtained at the B3LYP/def2-TZVPPD/MWB52(Eu)/CPCM level of theory.

Atom	g_j	ρ
O(1)	0.773	0.0626
O(2)	0.729	0.0612
O(3)	0.907	0.0598
O(4)	0.721	0.0637
O(5)	0.667	0.0613
O(6)	0.713	0.0625
O(7)	0.880	0.0630
O(8)	0.795	0.0613

Table S9. Ligand field parameters (B^k_q) for the ground state geometry of $[Eu(tta)_4]^-$ at the B3LYP/def2-TZVPPD/MWB52(Eu)/CPCM level of theory.

Parameter	Complex value / cm^{-1}	Absolute value / cm^{-1}
B^2_0	$252.753 + 0i$	252.753
B^2_1	$46.871 + 90.591i$	101.998
B^2_2	$187.569 + 17.174i$	188.292
B^4_0	$-71.226 + 0i$	71.226
B^4_1	$-55.806 + 291.216i$	296.515
B^4_2	$-166.341 - 115.791i$	202.674
B^4_3	$-48.235 + 229.811i$	234.819
B^4_4	$-157.047 - 205.127i$	258.342
B^6_0	$15.725 + 0i$	15.725
B^6_1	$-18.933 + 134.981i$	136.302
B^6_2	$-55.164 - 40.504i$	68.437
B^6_3	$-70.186 + 92.933i$	116.483
B^6_4	$-19.713 + 24.425i$	31.388
B^6_5	$-90.358 + 42.433i$	99.826
B^6_6	$-94.852 - 162.003i$	187.278

4f-4f $\langle \mathrm{f}||C^{(k)}||\mathrm{f}\rangle$ matrix elements can be analytically calculated: $\langle \mathrm{f}||C^{(2)}||\mathrm{f}\rangle = -1.366$, $\langle \mathrm{f}||C^{(4)}||\mathrm{f}\rangle = 1.128$, $\langle \mathrm{f}||C^{(6)}||\mathrm{f}\rangle = -1.270$

Table S10. Numerical values of the free Eu^{3+} ion $\langle \psi(SL)J||U^{(k)}||\psi(SL)J\rangle$ reduced matrix elements in the intermediate coupling scheme.

Level	$U^{(2)}$	$U^{(4)}$	$U^{(6)}$
$^7F_1$	0.3925	0	0
$^7F_2$	0.3162	−0.3493	0
$^7F_3$	0.1662	0.1612	0.1677
$^7F_4$	−0.1084	0.5330	−0.5938
$^7F_5$	−0.5235	0.4543	0.5677
$^7F_6$	−1.0965	−0.6276	−0.1749

If any reader wishes to obtain the value of the reduced matrix elements of another level for any lanthanide ion, we refer them to the tables of Nielson and Koster published in their 1963 book.

Supporting Information

Calculation of the Eu^{3+} photophysical parameters such as the $4f^n$-$4f^n$ intensity parameters ($\Omega_{2,4}$), the radiative rates (A_{rad}), non-radiative decay rates (A_{nrad}) and intrinsic quantum yields (ϕ_{Ln}^{Ln}):

The $^5D_0 \rightarrow {}^7F_1$ transition is magnetic-dipole allowed and practically insensitive to the chemical environment. Its radiative rate can be calculated in units of s^{-1} using the refractive index of the medium and the transition frequency through the equation (Eq. S1):

$$A_{01} = \frac{8\pi^3 e^2}{3(m_e c)^2} \langle {}^7F_1 || \boldsymbol{L} + 2\boldsymbol{S} || {}^5D_0 \rangle^2 \tilde{\nu}_{01}^3 n^3 = 0.313 \times 10^{-11} \tilde{\nu}_{01}^3 n^3 \quad \text{(S1)}$$

with m_e being the electron mass, e is the elementary charge, c is the speed of light, $\tilde{\nu}_{01}$ is the transition centroid in wavenumbers, and n is the refractive index of the medium. Given that a transition area in terms of counts per second is proportional to the transition intensity $I_{J'J} = N_{J'} A_{J'J}$ (where $N_{J'}$ is the population of the emitting level), we can take the ratio of the electric dipole character $^5D_0 \rightarrow {}^7F_{2,4}$ transitions areas to the $^5D_0 \rightarrow {}^7F_1$ to obtain their spontaneous emission coefficient and the total radiative rate (Eq. S2 and S3):

$$\frac{S_{0J}}{S_{01}} = \frac{I_{0J}}{I_{01}} = \frac{N_0 A_{0J}}{N_0 A_{01}} \rightarrow A_{0J} = A_{01} \frac{S_{0J}}{S_{01}} \quad \text{(S2)}$$

$$A_{\text{rad}} = \sum_J A_{0J} \quad \text{(S3)}$$

The $4f^n$-$4f^n$ intensity parameters Ω_λ are then calculated using the Judd-Ofelt and Dynamic coupling theories (Eq. S4) [14–16,26,38]:

$$A_{0J} = \frac{4e^2\omega^3}{3\hbar c^3} \frac{n(n^2+2)^2}{9} \Omega_J \left\langle {}^7F_J \middle|\middle| U^{(J)} \middle|\middle| {}^5D_0 \right\rangle^2 \quad \text{(S4)}$$

Where ω is the transition angular frequency, $\hbar$ is the reduced Planck constant, Ω_J is the 4f-4f intensity parameter, and $\left\langle {}^7F_J \middle|\middle| U^{(J)} \middle|\middle| {}^5D_0 \right\rangle$ is the reduced matrix element of the multi-electron unitary tensor operator of rank J. With the values of the radiative rates and the luminescence decay time, one can calculate the non-radiative rate (A_{nrad}) and the intrinsic quantum yield of the Eu^{3+} ion (ϕ_{Ln}^{Ln}) using the following equation (Eq. S5):

$$\phi_{Ln}^{Ln} = \tau \sum_J A_{0J} = \frac{A_{\text{rad}}}{A_{\text{rad}} + A_{\text{nrad}}} \quad \text{(S5)}$$

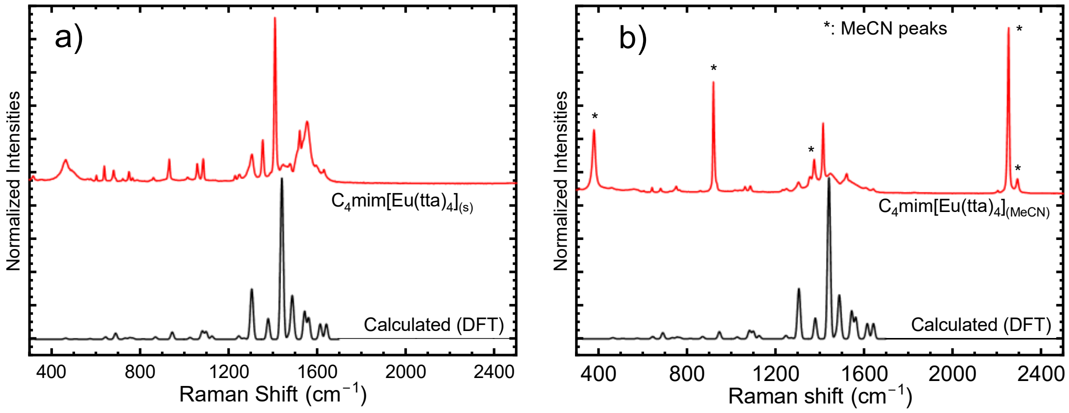

Figure S5. Theoretical Raman spectrum calculated for the $[Eu(tta)_4]^-$ complex at the B3LYP/def2-TZVPPD/MWB52(Eu)/CPCM level of theory (black line) compared with the experimental Raman spectra of the $C_4mim[Eu(tta)_4]$ (red line) in the (a) solid state and (b) MeCN solution.
